# A bioinformatics investigation into the pharmacological mechanisms of the effect of the Yinchenhao decoction on hepatitis C based on network pharmacology

**DOI:** 10.1186/s12906-020-2823-y

**Published:** 2020-02-12

**Authors:** Jingyuan Zhang, Xinkui Liu, Jiarui Wu, Wei Zhou, Jinhui Tian, Siyu Guo, Shan Shan Jia, Ziqi Meng, Mengwei Ni

**Affiliations:** 1 Department of Clinical Chinese Pharmacy, School of Chinese Materia Medica, Beijing University of Chinese Medicine, No. 11 of North Three-ring East Road, Chao Yang District, Beijing, China; 2 Evidence-Based Medicine Center, School of Basic Medical Sciences, Lanzhou University, 222 Tianshui South Road, Lanzhou City, China

**Keywords:** Yinchenhao decoction, Hepatitis C, Network pharmacology, Bioinformatics

## Abstract

**Background:**

Globally, more than 170 million people are infected with hepatitis C virus, a major cause of cirrhosis and hepatocellular carcinoma. The Yinchenhao Decoction (YCHD) is a classic formula comprising three herbal medicines. This decoction have long been used in China for clinically treating acute and chronic infectious hepatitis and other liver and gallbladder damp heat-accumulation disorders.

**Methods:**

In this study, we identified 32 active ingredients and 200 hepatitis C proteins and established a compound-predicted target network and a hepatitis C protein–protein interaction network by using Cytoscape 3.6.1. Then, we systematically analyzed the potential targets of the YCHD for the treatment of hepatitis C. Finally, molecular docking was applied to verify the key targets. In addition, we analyzed the mechanism of action of the predicted targets by the Kyoto Encyclopedia of Genes and Genomes and gene ontology analyses.

**Results:**

This study adopted a network pharmacology approach, mainly comprising target prediction, network construction, module detection, functional enrichment analysis, and molecular docking to systematically investigate the mechanisms of action of the YCHD in hepatitis C. The targets of the YCHD in the treatment of hepatitis C mainly involved PIK3CG, CASP3, BCL2, CASP8, and MMP1. The module and pathway enrichment analyses showed that the YCHD had the potential to influence varieties of biological pathways, including the TNF signaling pathway, Ras signaling pathway, PI3K-Akt signaling pathway, FoxO signaling pathway, and pathways in cancer, that play an important role in the pathogenesis of hepatitis C.

**Conclusion:**

The results of this study preliminarily verified the basic pharmacological effects and related mechanisms of the YCHD in the treatment of hepatitis C.

## Background

In Asian countries, Traditional Chinese medicine (TCM), which holds an irreplaceable position, has a long history of application in the prevention and treatment of diseases [1]. One among the classic TCM formulae derived from the *Treatise on Exogenous Febrile Disease* (Shanghan Lun), the Yinchenhao Decoction (YCHD) comprises Yinchen (*Artemisiae Scopariae Herba*), Zhizi (*Gardeniae Fructus*), and Dahuang (*Rhei Radix et Rhizoma*) and has found widespread application in the treatment of jaundice for more than 2000 years [2]. Pharmacological studies have shown YCHD can not only be used to treat pancreatic carcinoma, liver injury, liver fibrosis, cirrhosis, nonalcoholic steatohepatitis, and cholestasis, but also plays a role in anti-inflammatory, anti-pathogenic microbial activity as well as has anticancer effects. Therefore, the YCHD formula has long been clinically used for acute and chronic infectious hepatitis and hepatobiliary conditions [3–5].

Hepatitis C infection results in acute and chronic hepatitis. The hepatitis C virus (HCV) is an enveloped RNA virus that is spread as a sexually transmitted and blood-borne infection that infects humans only, and primarily targets liver cells. The HCV evades innate and adaptive immunity and establishes chronic infections in 70% of infected cases. Cirrhosis develops in 20% of untreated patients, and hepatocellular carcinoma (HCC) occurs in a fraction of these patients. Hepatitis C is a global health problem, and an estimated 170 million individuals are chronically infected with hepatitis C virus (HCV) [6–8]. World Health Organization (WHO) plans to eliminate HCV infection by 2030. However, we would like to highlight two additional barriers to achieving elimination of HCV infection: the high prevalence of this infection in resource-poor settings and suboptimal prevention of primary and repeated infection. Currently, patients with hepatitis C are usually treated with ribavirin combined with ribavirin and pegylated interferon (PEG IFN) [9]. The antiviral activity of ribavirin and PEG IFN is based upon their ability to stimulate interferon stimulated genes (ISGs) that have endogenous antiviral activities. The shortcomings of ribavirin and PEG IFN therapy were significant, most importantly the poor tolerance and side effects of this regimen [10]. Therefore, we need to find drugs with better drug resistance and better economy to treat hepatitis C.

**Fig. 1:**
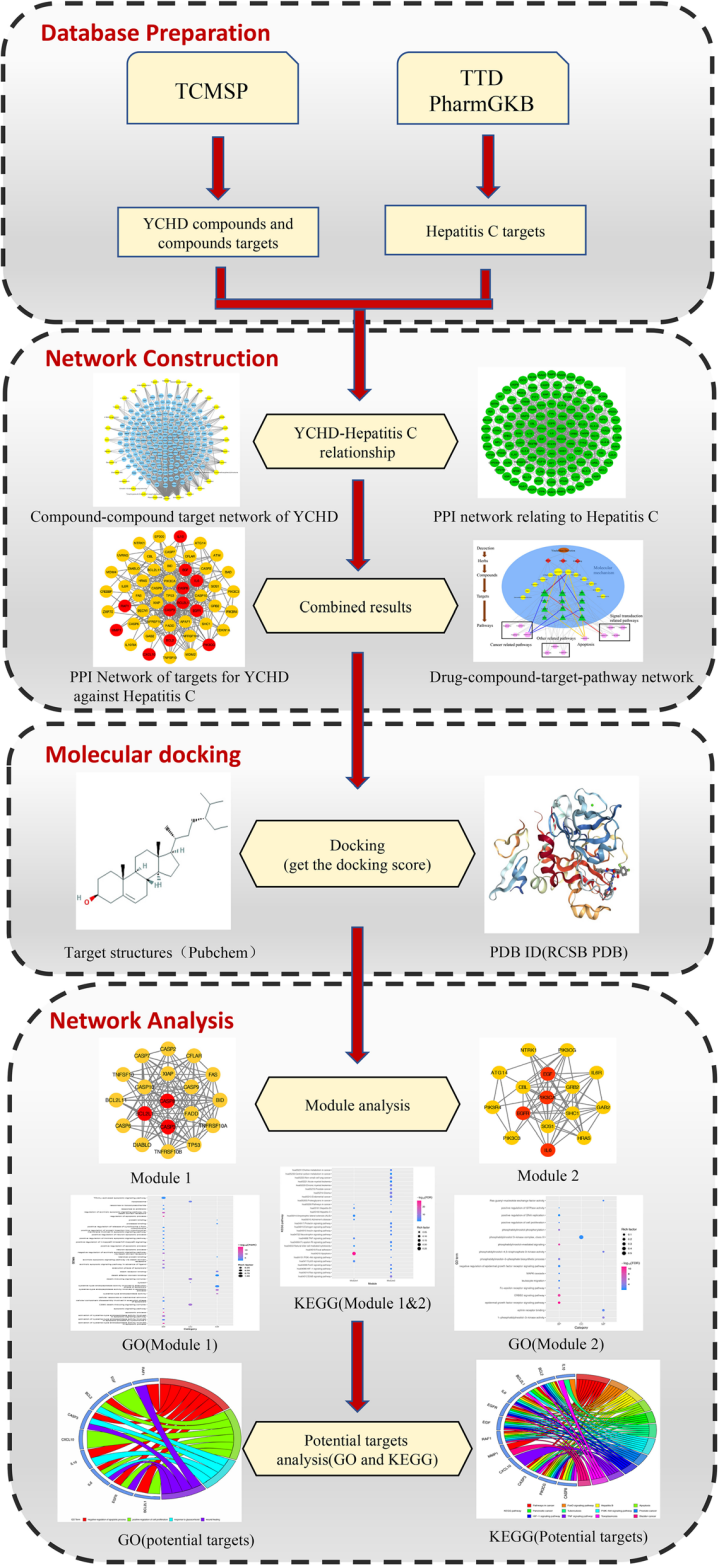
Workflow for Yinchenhao Decoction (YCHD) in the treatment of hepatitis C

Numerous studies on the treatment of viral hepatitis by the YCHD offer a good opportunity for effective data mining and network pharmacology research in this subject. Clinically, YCHD combined with IFN and ribavirin has shown significant clinical effects and a low incidence of adverse reactions in the treatment of patients with chronic HCV infection [11]. Network pharmacology – based on network and systems biology –has been extensively applied by TCM researchers. Because TCM exhibits therapeutic efficacy by the synergistic effects of multicomponent, multitarget, and multipathway mechanisms, it is relatively difficult to analyze the intricate mechanisms of TCM merely by using traditional experimental approaches [12, 13]. Network pharmacology shares the same guiding theory as TCM practice“parts can be understood only in its relation to the whole” [14–17]. This study provides a systematic method for mapping the mechanism of action of YCHD in the treatment of HCV infection and identifying potential protein targets that coordinate the abovementioned synergistic effects of TCM. We undertook this study to provide insight into the further in-depth development of the basic experimental research and clinical rational application of YCHD. The study flowchart of this network pharmacology-based study of YCHD is shown in Fig. 1.

## Methods

### Data preparation

#### Herbal compounds and compound targets in YCHD

To collect the herbal compounds of YCHD, we utilized the Traditional Chinese Medicine Systems Pharmacology Database [18, 19] (TCMSP, http://lsp.nwu.edu.cn/) – a unique system pharmacology platform devised for Chinese herbal medicines. All herbal compounds used in YCHD were retrieved on searches by using “Yinchen”, “Zhizi”, and “Dahuang” as the keywords. Oral bioavailability (OB) is an important indicator that determines whether oral drugs can overcome several obstacles, such as intestinal epithelium and wall, to reach the target site. Drugs with poor OB may show relatively low efficiency in entering the bloodstream and, thus, may have less beneficial therapeutic effects. To distinguish drugs from nondrugs, we adopted quantum mechanics-derived descriptors that depict the physical and partition properties of molecules to calculate the drug-likeness (DL) index; the DL index could help estimate the absorption, distribution, metabolism, and excretion (ADME) properties of the chemicals in the study drug [20]. Molecules with lower DL indices are considered less likely to be effective as drugs. In the current work, an DL value (0.18) and OB value (30%) were taken as cutoff limits to screen out the active compounds. Thereafter, we undertook a search for targets that had higher active compounds in the TCMSP. Finally, the information of compound targets was obtained.

#### Hepatitis C targets

Details on the human genes associated with hepatitis C infection were acquired from two resources: (1) the Therapeutic Target Database [21] (TTD; https://db.idrblab.org/ttd/) – a database of the known and explored therapeutic protein and nucleic acid targets, target disease, pathway information, and the corresponding drugs directed at each of these targets; and (2) the Pharmacogenomics Knowledgebase [22] (Pharm GKB; https://www.pharmgkb.org/) – a resource that collects, curates, and disseminates information about the impact of human genetic variations on drug responses.

#### Protein–protein interaction data

The protein–protein interaction (PPI) data were obtained from the Search Tool for the Retrieval of Interacting Genes (STRING) database [23, 24] (https://string-db.org), which provides information on predicted and experimental protein interactions. The prediction method of this database derives from the experiments, databases, and text mining of the neighborhood, gene fusion, cooccurrence, and co-expression. Furthermore, the database defines PPI with confidence ranges for data scores (low confidence: scores > 0.15; medium > 0.4; and high: > 0.7). In the present study, PPIs with combined scores higher than 0.7 were selected for further research.

### Network construction

Network construction was done as follows: (1) the compound–compound target network was established by connecting the herbal compounds and their corresponding targets; (2) a PPI network of hepatitis C targets was constructed by linking the known hepatitis C-related targets and other human proteins that interacted with them; (3) a PPI network of targets for YCHD against hepatitis C was built by intersecting the two networks of (1) and (2); and (4) a drug–compound–target–pathway network was established.

All the networks were visualized utilizing Cytoscape [25] (ver. 3.6.1; http://cytoscape.org/). Cytoscape is an open-source bioinformatics analysis software for constructing molecular interaction networks composed of proteins, genes, drugs, and other interactions for visual browsing and analysis. The topological features of interaction networks were evaluated by calculating three indices – degree, betweenness centrality, and closeness centrality – with a Cytoscape tool Network Analyzer [26–28]. The degree is defined as the number of edges to node i; betweenness is used to describe the number of shortest paths between pairs of nodes that run through node i; and closeness stands for the inverse of the sum of the distances from node i to other nodes. The higher the three quantitative values of a node are, the greater the importance of the node in the network.

### Molecular docking

The Research Collaboratory for Structural Bioinformatics Protein Data Bank [29] (RCSB PDB; https://www.rcsb.org) – the US data center for the global PDB archive – serves thousands of Data Depositors in the Americas and Oceania and makes available 3D macromolecular structure data free of charge. SystemsDock [30] (http://systemsdock.unit.oist.jp/iddp/home/index) is a web server for network pharmacology-based prediction and analysis, and it applies high-precision docking simulation and molecular pathway map to comprehensively characterize ligand selectivity and to illustrate how a ligand acts on a complex molecular network. The docking score of systemsDock is a negative logarithm of the experimental dissociation/inhibition constant (pKD/pKi), which can directly indicate the binding strength [31]. The PubChem Compound Database [32] (https://pubchem.ncbi.nlm.nih.gov) contains validated chemical depiction information provided to describe the components in a PubChem Substance. The structures stored within PubChem Compounds are pre-clustered and cross-referenced by identity and similarity groups. The RCSB PDB database was used to retrieve and download the PDB ID of the potential target proteins, whereas the active compound was downloaded as a 2D structure file using the PubChem database. The abovementioned two files were jointly imported into the SystemsDock online platform for molecular docking verification.

### Module analysis

The Cytoscape plugin Molecular Complex Detection (MCODE) [33] was applied to analyze clustering modules in the PPI network. In addition, both gene ontology (GO) and the Kyoto Encyclopedia of Genes and Genomes (KEGG) enrichment analysis were conducted on the module through the Database for Annotation, Visualization and Integrated Discovery [34] (DAVID; https://david.ncifcrf.gov). The David database integrates multiple types of database resources, uses the improved Fisher’s exact test algorithm to undertake enrichment analysis on gene sets, and provides *P*-value and false discovery rate (FDR) for enriched analysis results. GO enrichment analysis illustrates the role of target proteins of TCM compounds in gene function, including the functions of three modules of biological processes, molecular functions, and cellular components [35]. The KEGG pathway enrichment analysis provides not only pathway functional annotations of a given gene set but also the pathway enrichment analysis [36]. Finally, GO and KEGG enrichment analyses were undertaken on the protein of the combined protein in network construction (3).

## Results

### Screening of active compounds and targets

The compounds were searched in the TCMSP and screened by OB of 30% or higher and DL of 0.18 or higher. In total, 44 compounds were searched from the YCHD; of these, 13 were from Yinchen, 15 from Zhizi, and 16 from Dahuang. 44 compounds were subjected to component target prediction and repeated component screening, and a total of 32 compounds and corresponding predicted targets were obtained. Additional file 1: Table S1 presents basic information of the 32 active compounds in the YCHD.

### YCHD compound–compound target network

**Fig. 2:**
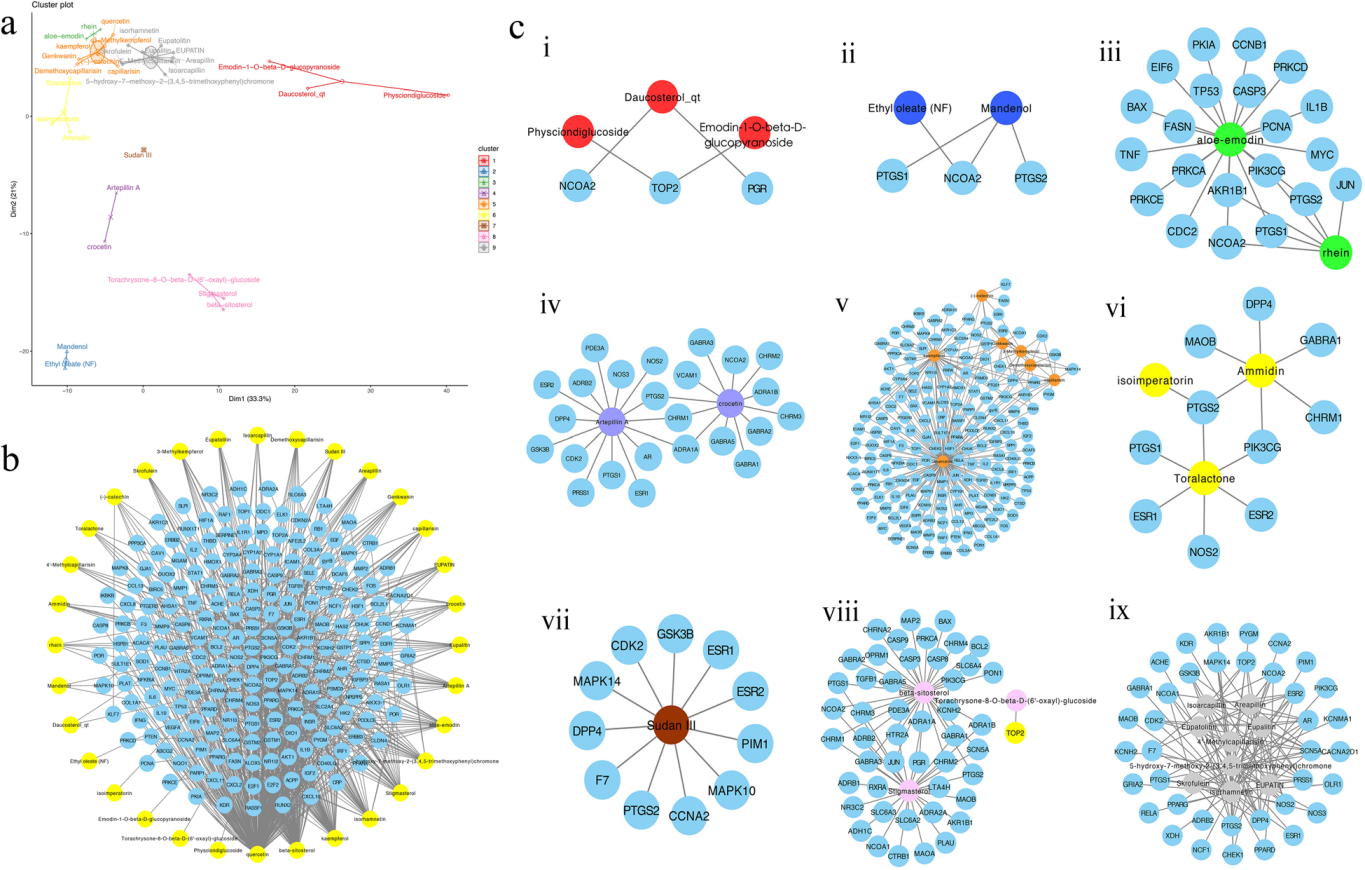
**a**: Cluster plot of 32 compounds, **b**: Compound–compound target network of YCHD (yellow and blue indicate the active ingredient and predicted target, respectively), **c**: Clustering compound target network (i) cluster 1; (ii) cluster 2; (iii) cluster 3; (iv) cluster 4; (v) cluster 5; (vi) cluster 6; (vii) cluster 7; (viii) cluster 8; and (ix) cluster 9. The red, dark blue, green, purple, orange, yellow, brown, pink, and gray circles represent the components clustered into the first, second, third, fourth, fifth, sixth, seventh, eighth, and ninth clusters, respectively. With the color corresponding to each compound cluster in Fig. 2a, the light blue circle indicates the predicted target of the compound

The details of the other 32 compounds in the YCHD are described in Fig. 2a, and these compounds were clustered into nine clusters. Then, Cytoscape was used to construct a compound–compound target network, which comprised 232 nodes (32 compound nodes and 200 target nodes) and 732 edges, where the yellow node represents the compound molecule (the active component) and the blue node represents the drug compound target (the predicted target). Each edge represents the interaction between the compound and the compound target (see Fig. 2b). In the network, the degree of a node indicates the number of routes it takes to connect to other nodes in the network. According to the topological properties of the network, nodes with a larger degree were screened for analysis. These nodes with more connected compounds or targets play a pivotal role in the network and may be key compounds or compound targets. In this network, the average target number of each compound is 6.25; therefore, there is an interaction between one compound and multiple targets in the YCHD. Furthermore, different compounds act on the same target together, which reflects the mechanism of interaction between multiple components and multiple targets of YCHD and conforms to the characteristics of TCM prescriptions. From the compound point of view, the number of the targets of 53.1% compounds were 10 or more, and the number of targets for seven compounds were 20 or higher. The top five compounds in degree were quercetin, beta-sitosterol, kaempferol, isorhamnetin, and stigmasterol, and they interacted with 292, 99, 58, 31, and 28 target proteins, respectively. With regard to the target, the top three in degree are PTGS2, NCOA2, and PTGS1, which can interact with 30, 21, and 21 compounds, respectively. The results of molecular clustering of 32 compounds were compared with each target point, and the results of the visualization are shown in Fig. 2c.

### PPT network of hepatitis C

**Fig. 3:**
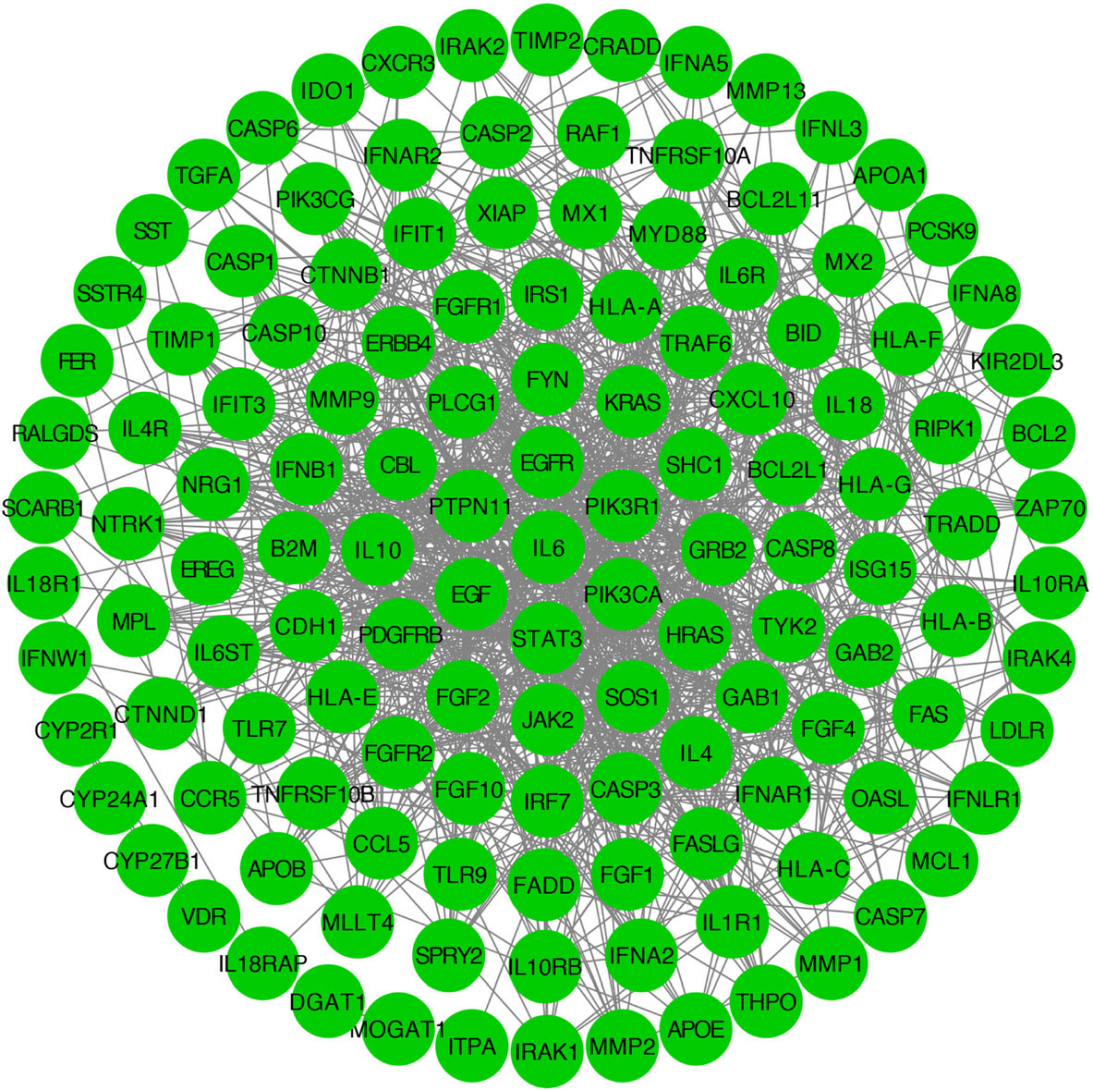
Protein–protein interaction network associated with hepatitis C

Additional file 1: Table S2 shows 39 primary proteins associated with hepatitis C that were retrieved from the TTD and Pharm GKB. The entire list of 39 proteins is shown in Additional file 1: Table S2; the interacting secondary proteins of these 39 proteins were searched in the STRING10.5 database, and a total of 98 secondary proteins associated with hepatitis C were identified. The PPI network of hepatitis C contains 137 proteins and 1063 PPIs associated with hepatitis C. Based on our findings, we constructed a hepatitis C-related PPI network by using Cytoscape 3.6.1 (Fig. 3).

### PPI network of targets for the YCHD against hepatitis C

**Fig. 4:**
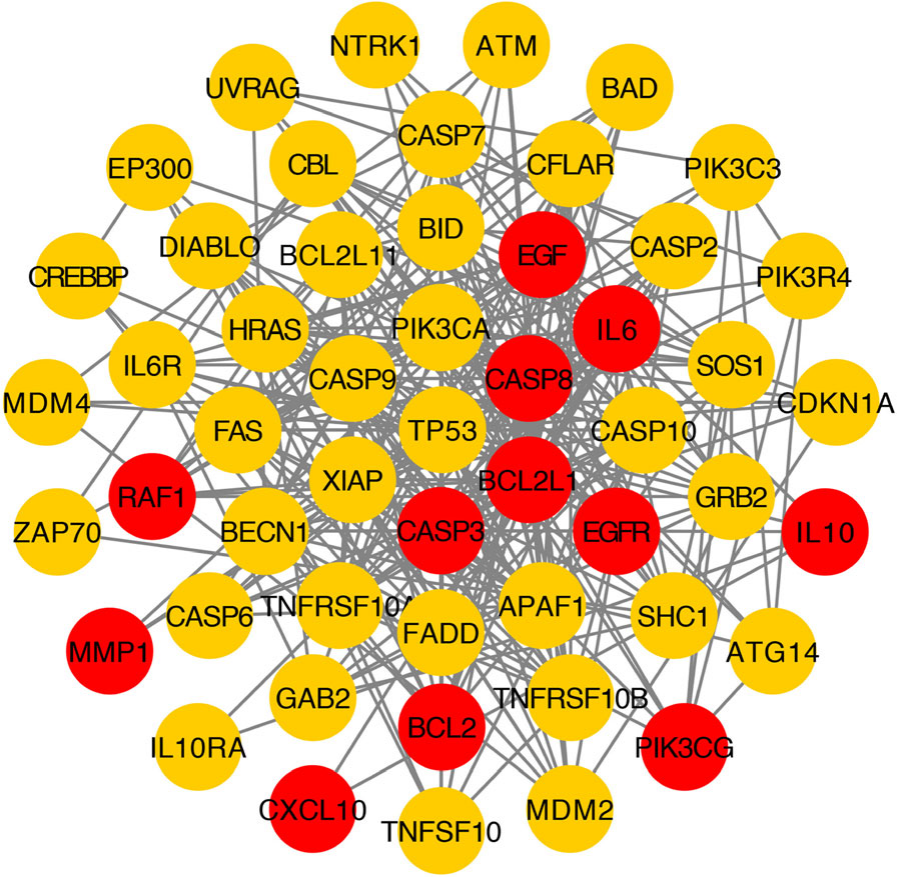
PPI Network of targets for Yinchenhao Decoction (YCHD) against hepatitis C. Red and orange represent the potential and other human target proteins, respectively, for YCHD in the treatment of hepatitis C

The combination of the YCHD compound–compound target network and the PPI network of hepatitis C can be used to obtain the potential target of the YCHD in the treatment of HCV infection. The principle of the connection between nodes is that when the predicted target of the active ingredient in the YCHD is the same as the target of HCV, the predicted target is linked to be a target of HCV. The same target is the potential target of active ingredients for the treatment of hepatitis C with the YCHD. By the Merge combined analysis, we obtained 12 targets: phosphatidylinositol-4,5-bisphosphate 3-kinase catalytic subunit, gamma isoform (PIK3CG), caspase-3 (CASP3), apoptosis regulator Bcl-2 (BCL2), caspase-8 (CASP8), interstitial collagenase (MMP1), C-X-C motif chemokine 10 (CXCL10), RAF proto-oncogene serine/threonine-protein kinase (RAF1), interleukin-6 (IL-6), pro-epidermal growth factor (EGF), interleukin-10 (IL-10), Bcl-2-like protein 1 (BCL2L1), and epidermal growth factor receptor (EGFR). The above potential target data were uploaded into the STRING database for the PPI network (Fig. 4). There are 52 nodes and 291 edges in the figure. Red and orange, respectively, represent the potential and other human target proteins of the YCHD in the treatment of hepatitis C. Orange represents other human target proteins of YCHD in the treatment of Hepatitis C.

### Molecular docking verification

Based on the combined results in Fig. 4, we obtained 12 potential targets for the treatment of hepatitis C. The molecular docking verification was carried out for these 12 abovementioned targets. The PDB IDs of these targets were respectively introduced into the SystemsDock, and these proteins were docked with the active components of the YCHD (Table 1). The PDB IDs of two of the targets are unavailable; therefore, they are not discussed in this study. The docking scores of most of them were larger than 5.52, which showed that they possessed good binding activity [37, 38]. This results provide a theoretical basis for further explanation of YCHD for the treatment of the Hepatitis C.

**Table 1:** Docking score of active ingredients with potential targets of Yinchenhao Decoction

Protein	PDB ID	2D Image	Test Compounds	Docking Score (pKD/pKi)
BCL2	2W3l		beta-sitosterol	6.818
			quercetin	5.928
			kaempferol	5.888
CASP8	1QTN		beta-sitosterol	5.013
			quercetin	6.375
IL6	1ALU		quercetin	6.688
EGF	1NQL		quercetin	6.185
IL10	2ILK		quercetin	6.642
CASP3	6BFJ		aloe-emodin	4.871
			beta-sitosterol	7.274
			quercetin	4.875
			kaempferol	4.856
PK3CG	3CSF		rhein	6.621
			aloe-emodin	6.967
			Ammidin	4.49
			quercetin	6.44
			Toralactone	6.866
			beta-sitosterol	7.696
			3-Methylkempferol	4.354
			kaempferol	6.694
			capillarisin	4.448
			isorhamnetin	4.491
			Demethoxycapillarisin	6.328
MMP1	1SU3		quercetin	6.323
			kaempferol	6.505
CXL10	1O7Y		quercetin	4.741
RAF1	3KUC		quercetin	6.464

### Module analysis

**Fig. 5:**
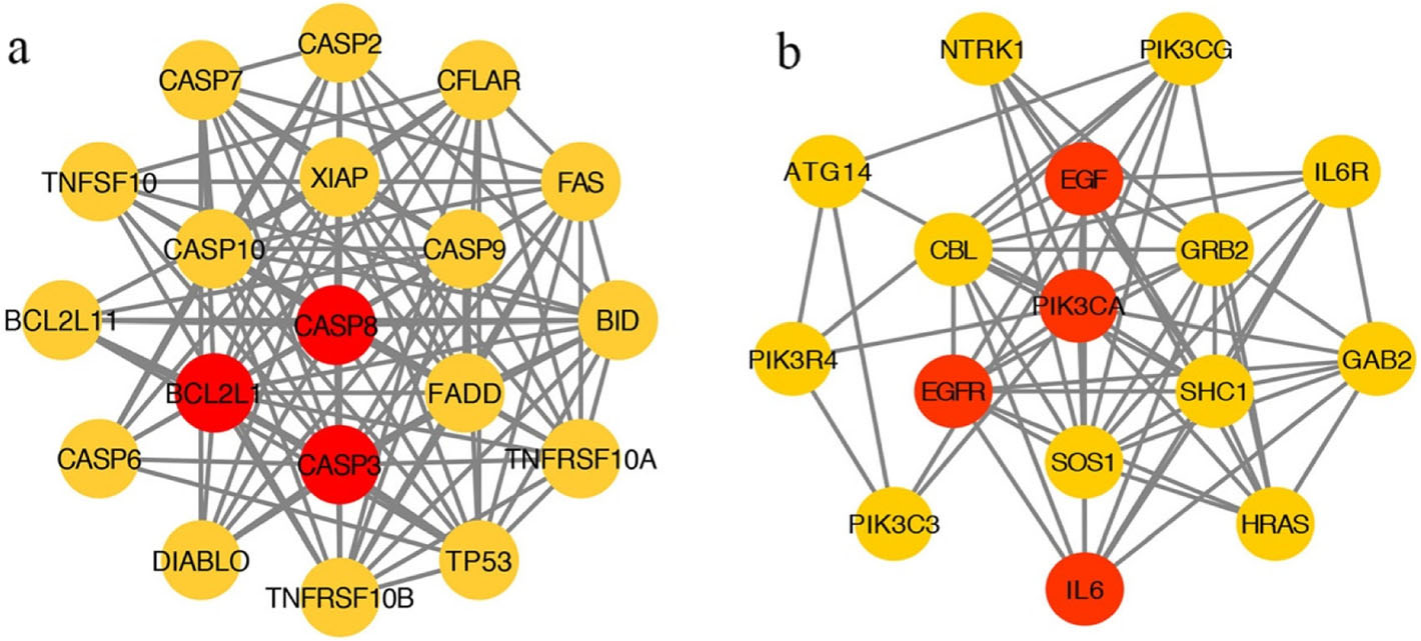
Module analysis: (**a**) Module 1 and (**b**) Module 2. Red and orange represent the potential and other human target proteins, respectively, for Yinchenhao Decoction in the treatment of hepatitis C

**Fig. 6:**
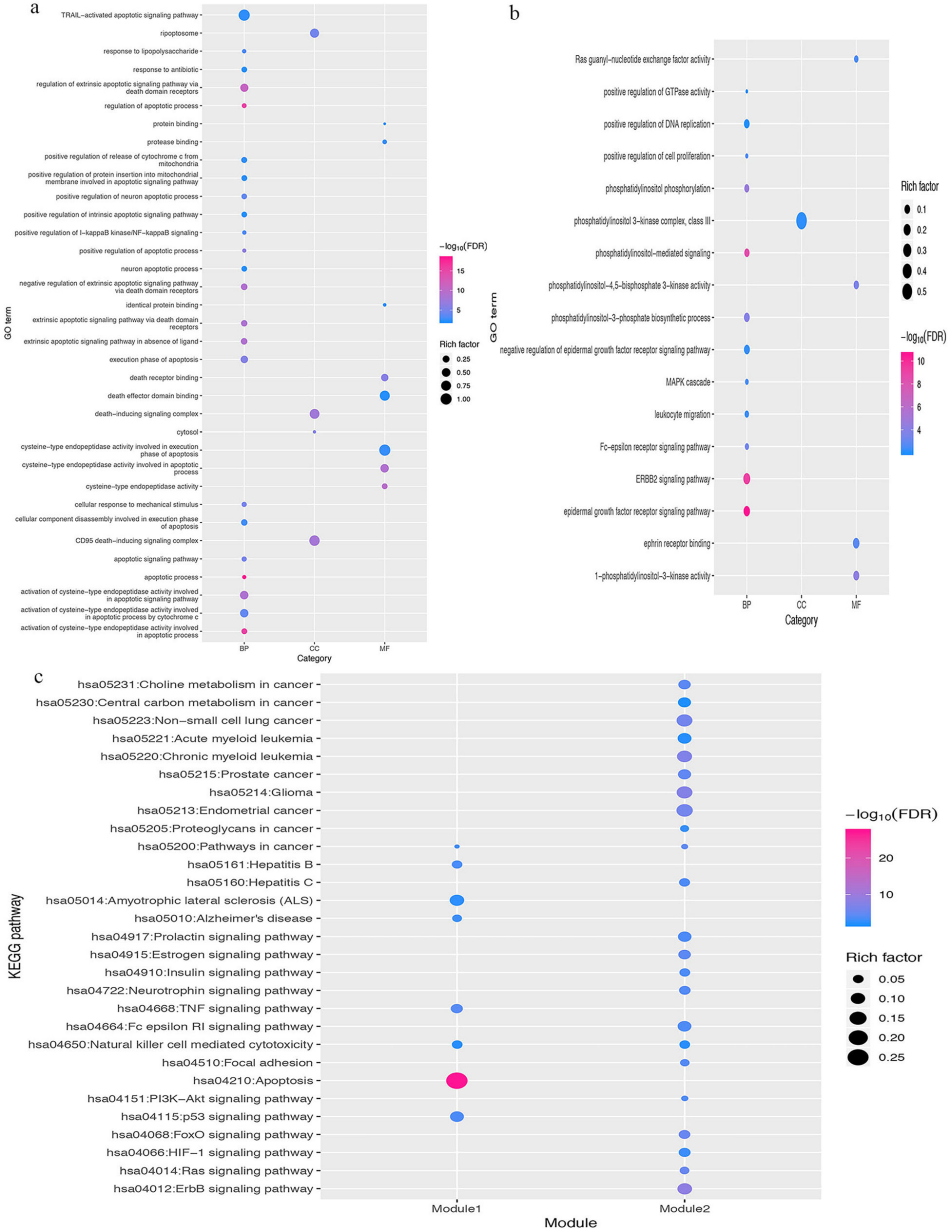
(**a**) Gene ontological (GO) analysis of Module 1; (**b**) GO analysis of Module 2; and (**c**) KEGG enrichment analysis of modules 1 and 2

MCODE is based on complex algorithms that cluster objects with similar properties. We clustered the data in the potential target PPI network of the YCHD in the treatment of hepatitis C, and obtained two modules (a: Module 1, score = 12.889; b: Module 2, score = 8.667, Fig. 5). Both GO and KEGG enrichment analyses were conducted on the data in this module, and the results are shown in Fig. 6 (detailed data information in the Additional file 1: Tables S3-S6).

The GO enrichment analysis of Module 1 obtained a total of 104 entries. With a *P* < 0.01 and FDR < 0.01, we obtained 35 entries: 23 items were related to biological process (BP), eight items to molecular function (MF), and four items to the cellular component (CC). The GO enrichment analysis of Module 2 obtained a total of 128 entries. With *P* < 0.01 and FDR < 0.01, we obtained 17 entries: 12 related to BP, four items to MF, and one entry related to the CC.

In Module 1, we screened eight pathways according to *P* < 0.01 and FDR < 0.01; these mainly included the apoptotic pathway (hsa04210); TNF signaling pathway (hsa04668); p53 signaling pathway (hsa04115), etc. In Module 2, we screened 23 pathways according to *P* < 0.01 and FDR < 0.01; these mainly included the ErbB signaling pathway (hsa04012); glioma pathway (hsa05214); chronic myeloid leukemia pathway (hsa05220); endometrial cancer pathway (hsa05213), etc.

### GO and KEGG enrichment analysis of potential targets

**Fig. 7:**
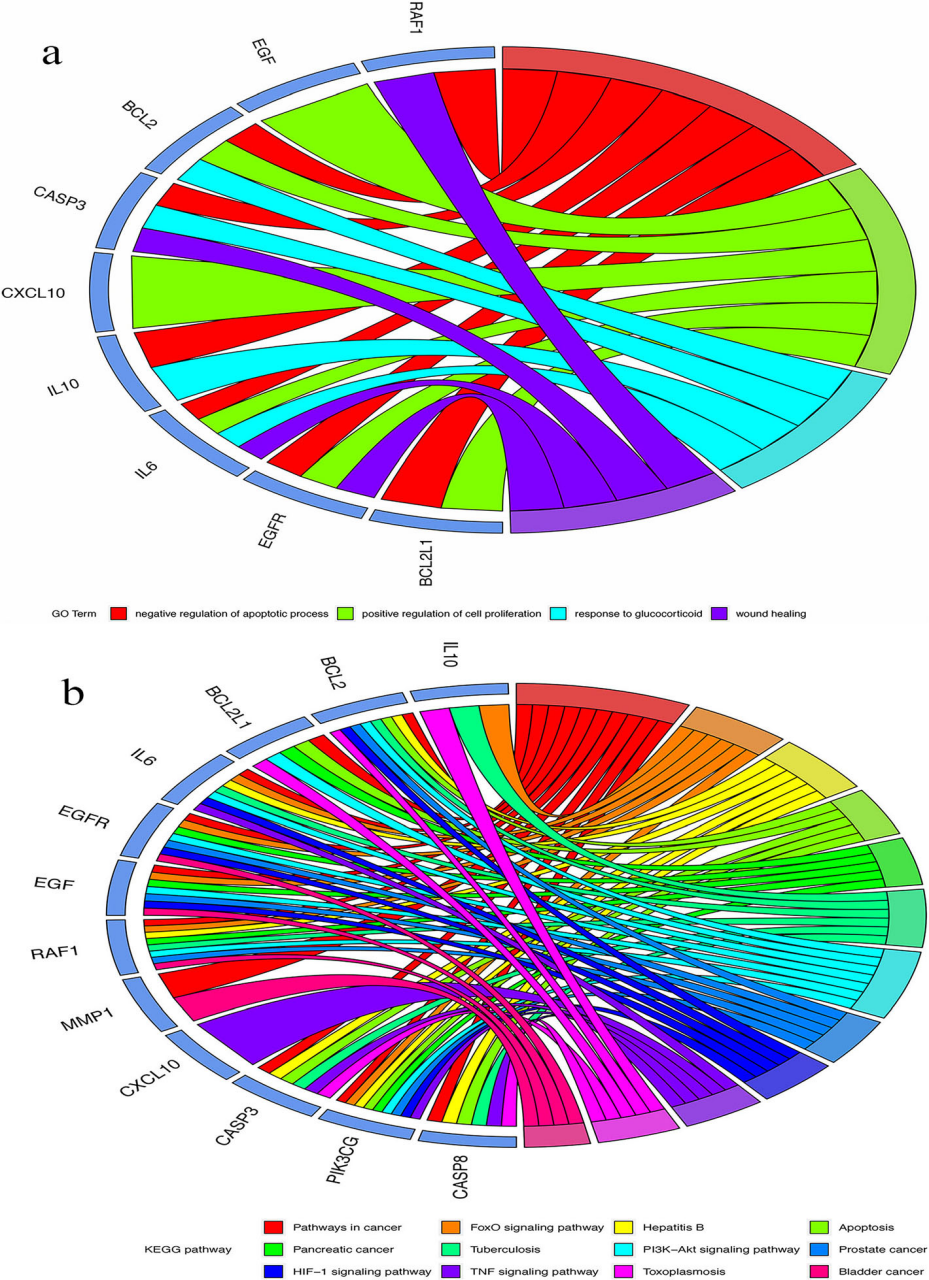
(**a**) Analysis of GO entries for potential targets of Yinchenhao Decoction (YCHD) in the treatment of hepatitis C. (**b**) Analysis of the KEGG pathway for potential targets of YCHD in the treatment of hepatitis C

Using the DAVID platform for GO functional enrichment analysis, we studied the role of 12 potential targets based on the combined results in gene function, and obtained 127 GO entries. Four GO entries were determined on the basis of the FDR (< 0.05; Fig. 7a and Additional file 1: Table S7). The four GO items are related to BP, namely: negative regulation of apoptotic process, positive regulation of cell proliferation, response to glucocorticoids, and wound healing.

The DAVID platform for KEGG enrichment analysis was used to research the role of 12 potential targets based on the combination results in pathway enrichment, and 59 pathways were obtained. Twelve pathways were determined by the FDR (< 0.05; Fig. 7b and Additional file 1: Table S8). Of these, the human disease-cancer overview pathway includes: pathway in cancer; human disease-specific cancer (e.g., pancreatic, bladder, and prostate cancers), human disease-viral infection (e.g., hepatitis B), human disease-bacterial infectious disease pathways (e.g., tuberculosis), human disease-parasitic infectious disease pathway (e.g., toxoplasmosis), environmental information processing-signal transduction pathways (e.g., PI3K-Akt signaling pathway, FoxO signaling pathway, HIF-1 signaling pathway, TNF signaling pathway), cellular processes-cell growth and death pathway (e.g., apoptosis).

### Integrated network construction

**Fig. 8:**
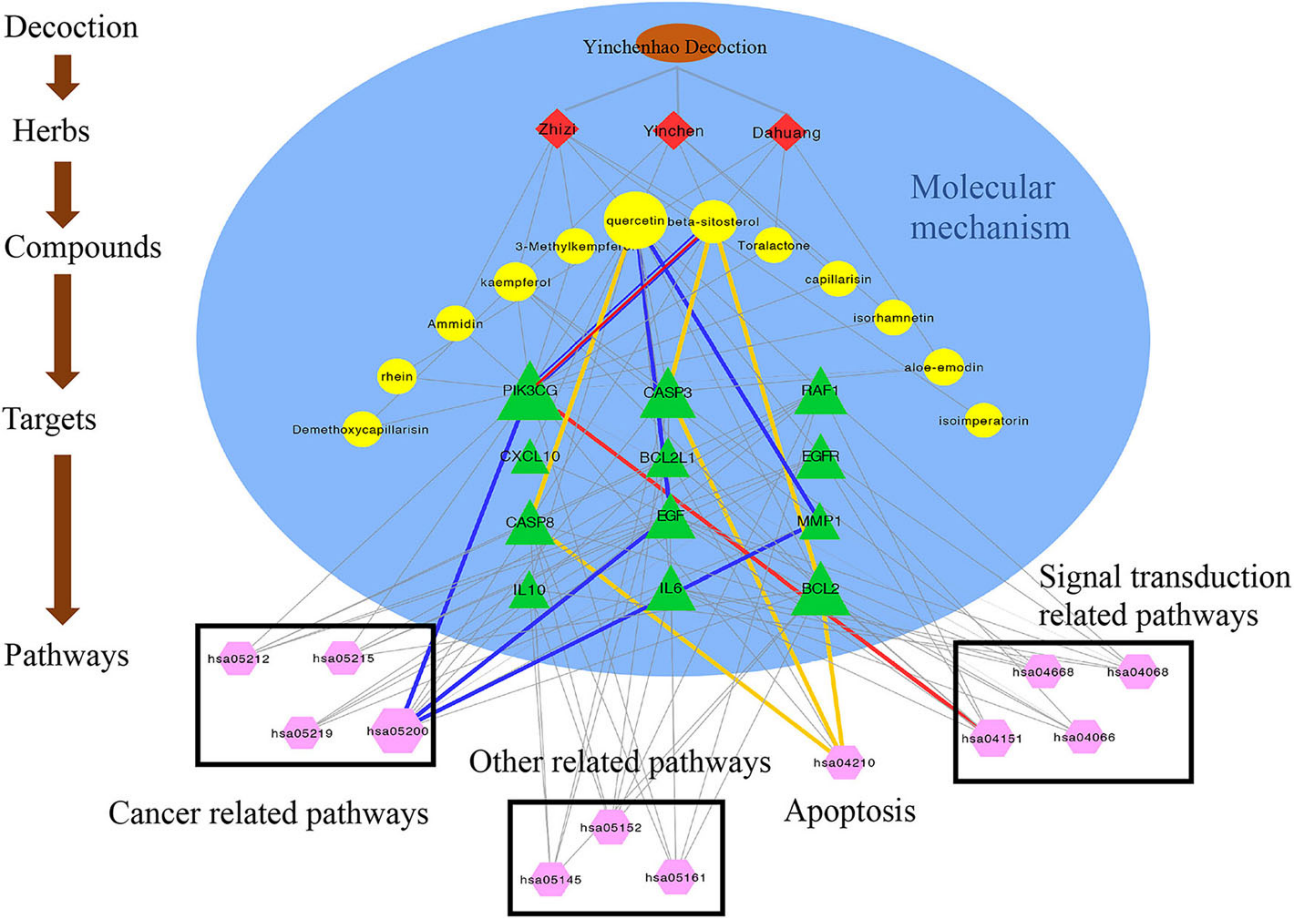
Drug–compound–target–pathway network (Brown indicates the YCHD; Red indicates the Yinchenhao Decoction; yellow indicates the compounds; green indicates the potential targets; and pink indicates the, pathways)

Based on our findings, we constructed a drug–compound–target–pathway network was constructed (Fig. 8). Brown indicates the YCHD; red indicates the drugs; yellow indicates the compounds; green indicates the potential targets; and pink indicates the pathways. The blue line is connected to the cancer-related pathway – the pathway with the highest degree of value and its key target and the component with the highest docking score. The orange line is linked to the key target in the apoptotic pathway and the compound with the highest docking score. The red line is connected to the signal transduction-related pathway – the channel with the highest degree of value and its key target and the component with the highest docking score.

## Discussion

In the present study, we applied a network pharmacology approach to predict, elucidate, and confirm the potential mechanisms of action of the YCHD on hepatitis C by integrating target prediction, network construction, and module analysis. The analysis of compound–compound target network and PPI network of compound targets displayed that PIK3CG, CASP3, BCL2, CASP8, and MMP1 might be the key targets of the YCHD in hepatitis C. The module analysis found that the YCHD has the potential to influence varieties of biological pathways that play an important role in the pathogenesis of hepatitis C, including the TNF signaling pathway, Ras signaling pathway, etc. Twelve pathways were obtained by KEGG pathway enrichment analysis, including those that involve the PI3K-Akt signaling pathway, FoxO signaling pathway, pathways in cancer, etc.

HCV infection is associated with various extrahepatic manifestations, which are correlated with poor outcomes and, thus, increase the morbidity and mortality of chronic hepatitis C (CHC) infection [39]. The HCV is the etiological agent of fatal hepatitis, causing millions of deaths worldwide [40]. The HCV is mainly transmitted through blood, and the target cells are hepatocytes, which are recognized as the main site of replication of HCV. The pathological changes of hepatitis C were similar to those of hepatitis A, B, and D; the incidence of lymphocyte aggregation, bile duct injury, and steatosis in the portal area in HCV infection was significantly higher than that of HBV infection. Hepatocyte damage in CHC is not a direct cell damage effect of HCV, but instead is achieved by immune mediated or similar mechanisms. HCV can affect the host’s immune function in several different ways, allowing the virus to continue to replicate in the host, leading to CHC. At present, the most effective anti-HCV drug treatment in clinical practice is PEG-IFN alpha combined with ribavirin; however, some patients cannot generate a sustained virological response [41]. The YCHD has the effect of clearing away heat and dampness, undertakes detoxification, jaundice, and is the main method for clinical treatment of damp heat and jaundice, and has a long history of use in medication [11]. In order to study the mechanism of the YCHD in the treatment of hepatitis C, we relied on the systematic pharmacological analysis platform of the TCMSP to study the active compounds of the three TCM agents (Yinchen, Zhizi, and Dahuang) in the YCHD. Simultaneously, the compound–compound target network, PPI network related to hepatitis C, and PPI network of targets for the YCHD against hepatitis C were constructed and the direct interaction between the active ingredient and target as well as target and pathway was analyzed. This provided a reference for the pharmacological mechanism of the YCHD in the treatment of hepatitis C – “multicomponent–multitarget–multipathway”.

The top five degrees of the potential targets for the YCHD in the treatment of hepatitis C include PIK3CG, CASP3, BCL2, CASP8, and MMP1. Apoptosis – a form of cell damage in various liver diseases – is worthy of study with regard to its role and significance in the pathogenesis and progression of viral hepatitis [42, 43]. Studies have shown that hepatocyte apoptosis is an effective defense mechanism against infection by pathogenic microorganisms and the spread of infection; moreover, it plays an important regulatory role in the malignant transformation of hepatocytes [44]. The caspase family plays an important role in hepatocellular apoptosis. The Caspase family is the initiator and executor of cell apoptosis. The activation of CASP8 is the first step in the caspase cascade. As a central molecule for the induction and downstream execution of apoptosis, CASP3 is involved in the condensation of cell chromatin and the activation of nucleases, which promotes irreversible apoptosis and has the unique advantages of inducing apoptosis, caspase activation, and degradation of its substrate, which then produces end-effect events that cause characteristic morphological changes in hepatic cells [45]. Previous studies have shown that the severity of liver damage in CHC infection is associated with the degree of hepatocyte apoptosis [46, 47]. However, the contribution of apoptosis or the molecular mechanisms that cause liver cell damage during HCV infection have not yet been clearly defined. Recently, death receptor DR4/DR5 has been recognized as a specific mediator of HCV-induced hepatocyte apoptosis [48]. Thus, we sought to evaluate critical determinants, particularly CASP8 activation, of the DR4/DR5-dependent apoptotic signaling pathway induced by HCV infection. Researchers propose that HCV infection promotes CASP8 activity, followed by activation of CASP3, as evidenced by suppression of DR4/DR5-mediated PARP cleavage via translocation of the CASP8-truncated Bid. Furthermore, we can suggest that the mechanism of the YCHD in the treatment of hepatitis C may be related to the induction or activation of CASP3, CASP8, and promotion of hepatocyte apoptosis. BCL-2 is a key regulator of apoptosis and exhibits anti-apoptotic activity [49, 50]. We compared the statistical relationship between the *BCL2* gene (Ala43Thr) single-nucleotide polymorphism and growth hormone (GH1) levels in Egyptian HCV genotype-4 patients, both before and after treatment with PEG IFN plus ribavirin. The results showed that patients with HCV genotype-4 with normal GH1 concentration and the BCL-2/43Ala genotype could successfully achieve a response to IFN therapy [51]. We undertook to investigate the expression and relationship of the HCV core proteins – P21 and Bcl-2 – in tissues of patients with hepatitis C and cirrhosis; the positive expression of HCV core protein and mutant P21 is mainly located in the nucleus; for Bcl-2, the positive expression was mainly located in the cytoplasm; furthermore, the positive expression rate of P21 was only 13%, and the positive expression rate of Bcl-2 was 95.7%. These results indicate that the expression of the three proteins is correlated, and the HCV core protein may promote the expression of Bcl-2 protein and inhibit the expression of P21 protein. Therefore, we suggested that the YCHD can maintain the sustained expression of BCL-2 and induce apoptosis, such that the clinically effective regimen of PEG and ribavirin produces a sustained response. MMP1 can decompose interstitial collagen, participate in the decomposition of extracellular matrix in normal physiological processes, and play a vital role in tissue repair and remodeling [52]. The overexpression of MMP1 reduces the number of activated hepatic stellate cells (HSCs) that are activated during transient overexpression in the liver and reduces liver fibrosis. These results indicate the protective effect of MMP1 during liver injury [53–55]. Although HCV infection in hepatocytes triggers key fibrotic factors in HSCs, we can show that the YCHD can promote the expression of MMP1, reduce the occurrence of liver fibrosis, and control further progression of hepatitis C infection. In summary, it is speculated that the active ingredients in the YCHD may treat hepatitis C by regulating these key targets. PIK3CG encodes a protein of the PI3/PI4 kinase family and is an important regulator of extracellular signaling; thus, it plays an important role in maintaining the structural and functional integrity of epithelial cells. The PIK3CG is a secondary protein obtained by the protein interaction network for the hepatitis C disease target. There is no related research to show the PIK3CG is closely related to hepatitis C. Therefore, it is necessary to further explore the mechanism of the YCHD in modulating hepatitis C by regulating PIK3CG.

In order to illustrate the role of the YCHD in the gene function and signaling pathway, this study undertook a GO functional enrichment and KEGG pathway analysis on the potential targets. The GO function enrichment analysis found that the YCHD is mainly reflected in biological processes, primarily in the negative regulation of the apoptotic process, active regulation of cell proliferation, response to glucocorticoids, and wound healing. In the enrichment of the KEGG pathway of the gene, 12 pathways were obtained; thus, we infer the YCHD is a treatment of hepatitis C through multicomponent action on these signaling pathways. HCV infection is the direct cause of CHC, and persistent inflammation can lead to liver fibrosis, sclerosis, and even liver cancer. Inflammation is usually associated with abnormal cellular signaling pathways. After viral infection, viral proteins interfere with the normal signal transduction pathways of host cells, causing abnormal expression of pro-inflammatory and inflammation-related molecules, leading to inflammation [56]. Taking PI3K-AKT as an example, in HCV-infected patients, impaired T-cell responses are associated with persistent infection, and myeloid-derived suppressor cells (MDSCs) play a key role in suppressing T-cell responses. Patients with HCV infection regulate the production of monocyte-MDSCs in monocytes via the PI3K pathway and autocrine cytokines, thereby reducing inflammatory effects [57]. Persistent HCV infection appears to trigger an episode of immune depletion that may contribute to the persistence of the virus in the host, ultimately leading to HCC. Peripheral blood mononuclear cells from patients with CHC were studied to determine the spontaneous recruitment of cellular reactive oxygen species (cROS) as well as immunomodulatory and depletion markers, relative to healthy controls. The study found spontaneous onset of apoptosis signals and T-cell failure in CHC disease [58]. We suggest that the YCHD can regulate the apoptotic pathway through BCL-2, CASP3, and other targets, thereby promoting the occurrence of apoptosis. It can be seen that the results of this study are consistent with most existing studies. However, the regulation of the YCHD on other related target pathways needs further study. Most genes are enriched in cancer-related pathways, including pathways in pancreatic, bladder, and prostate cancer. HCV infection is recognized as one of the major risk factors for the induction of liver cancer, especially HCC. In recent years, the proportion of HCV-related liver cancer has increased [59]. It is concluded that the mechanism of action of the YCHD in the treatment of hepatitis C may be related to the action of cancer-related pathways and the inhibition of progression to cancer. Through pathway enrichment analysis of modules 1 and 2, most of the signaling pathways were found to be involved in apoptosis and signal transduction. This is consistent with the analysis of key targets.

## Conclusion

In the present study, we applied a network pharmacology approach to predict, elucidate, and confirm the potential mechanisms of the YCHD on hepatitis C by integrating target prediction, network construction, and module analysis. Firstly, a total of 200 targets affected by 32 bioactive compounds in the YCHD were obtained; these demonstrated a synergistic treatment strategy of TCM characterized by multicomponent, multitarget, and multipathway mechanisms. Secondly, the analysis of compound–compound target network and the PPI network of compound targets displayed that PIK3CG, CASP3, BCL2, CASP8, and MMP1 might be the key targets of the YCHD associated with hepatitis C. Thirdly, it can be seen by molecular docking that the active ingredient in the YCHD has strong binding activity to two potential targets and has good binding activity to most potential targets. The KEGG enrichment analysis of potential targets indicates that the therapeutic effect of the YCHD in hepatitis C may be related to various biological pathways; for example: pathways in cancer, PI3K-Akt signaling pathway, FoxO signaling pathway, tuberculosis, apoptosis, pancreatic cancer, prostate cancer, HIF-1 signaling pathway, TNF signaling pathway, toxoplasmosis, bladder cancer, etc. We confirmed the molecular mechanism of the YCHD in preventing and treating hepatitis C, providing the preliminary information and basis for further exploration of its mechanism of action; moreover, it provides a reference for studying the more complex mechanism of action of Chinese herbal compounds.

## Supplementary information

**Additional file 1:** **Table S1.** Information on 44 active compounds of YCHD. **Table S2.** List of 39 proteins related to hepatitis C. **Table S3.** Module 1 GO enrichment entry. **Table S4.** Module 2 GO enrichment entry. **Table S5.** Module 1 KEGG enrichment entry. **Table S6.** Module 2 KEGG enrichment entry. **Table S7.** List of GO enrichment results to the YCHD potential target associated with hepatitis C. **Table S8.** List of pathway enrichment results to the YCHD potential target associated with hepatitis C

## Data Availability

The datasets used and/or analyzed during the current study are available from the corresponding author upon reasonable request.

## Abbreviations

DAVID: Database for Annotation, Visualization and Integrated Discovery
FDR: False discovery rate
GO: Genome Ontology
KEGG: Kyoto Encyclopedia of Genes and Genomes
OMIM: Online Mendelian Inheritance in Man
PEG IFN: Pegylated interferon
PharmGKB: Pharmacogenomics Knowledge Base
PDB: Protein Data Bank
PPI network: Protein-protein interaction network
STRING: Search Tool for the Retrieval of Interacting Genes
TCM: Traditional Chinese medicine
TTD: Therapeutic Target Database
YCHD: Yinchenhao Decoction
