# Supplementary material for: A bioinformatics investigation into the pharmacological mechanisms of the effect of the Yinchenhao decoction on hepatitis C based on network pharmacology
Source: BMC Complement Med Ther. 2020 Feb 12;20:50. doi: 10.1186/s12906-020-2823-y (PMC7076901; doi:10.1186/s12906-020-2823-y)
Supplement: Supplementary file 1 — Additional file 1: Table S1. Information on 44 active compounds of YCHD. Table S2. List of 39 proteins related to hepatitis C. Table S3. Module 1 GO enrichment entry. Table S4. Module 2 GO enrichment entry. Table S5. Module 1 KEGG enrichment entry. Table S6. Module 2 KEGG enrichment entry. Table S7. List of GO enrichment results to the YCHD potential target associated with hepatitis C. Table S8. List of pathway enrichment results to the YCHD potential target associated with hepatitis C [file 12906_2020_2823_MOESM1_ESM.docx]

Table S1**:** Information on 44 active compounds of YCHD

| ID | Molecule Name | Structure | OB% | DL | Source |
| --- | --- | --- | --- | --- | --- |
| MOL002235 | EUPATIN | 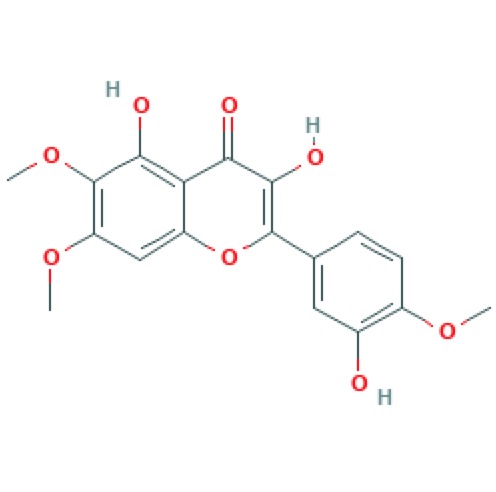 | 50.8 | 0.41 | *Rhei Radix et Rhizoma* |
| MOL002259 | Physciondiglucoside | 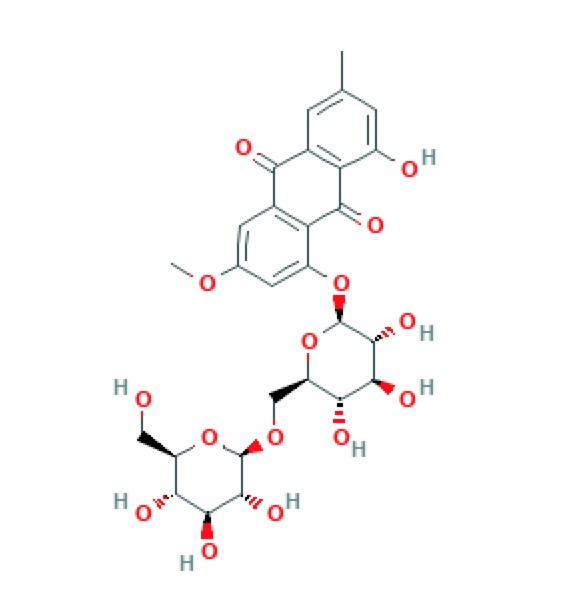 | 41.65 | 0.63 | *Rhei Radix et Rhizoma* |
| MOL002268 | rhein | 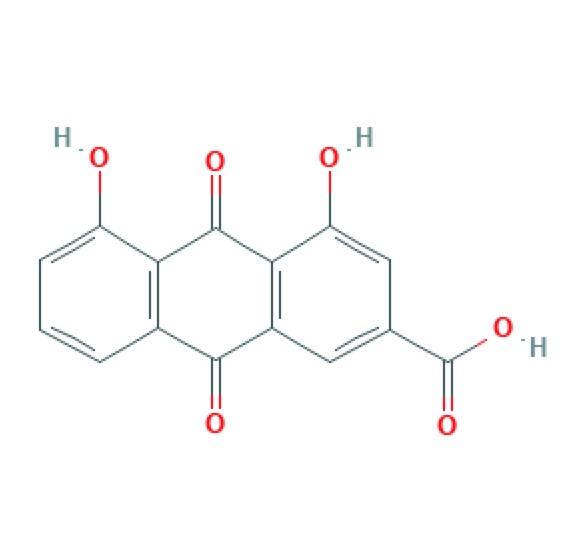 | 47.07 | 0.28 | *Rhei Radix et Rhizoma* |
| MOL002280 | Torachrysone-8-O-beta-D-(6'-oxayl)-glucoside | 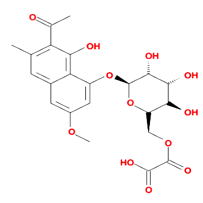 | 43.02 | 0.74 | *Rhei Radix et Rhizoma* |
| MOL002281 | Toralactone | 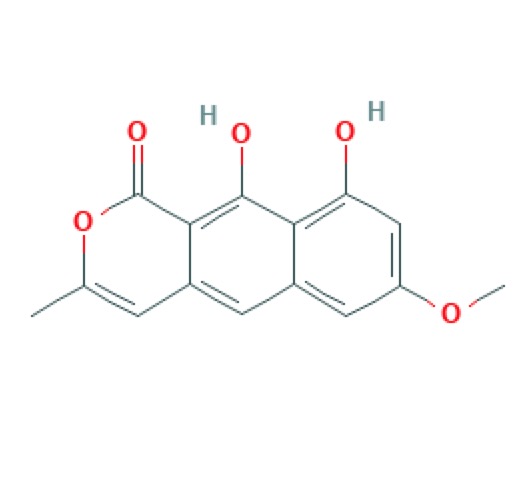 | 46.46 | 0.24 | *Rhei Radix et Rhizoma* |
| MOL002288 | Emodin-1-O-beta-D-glucopyranoside | 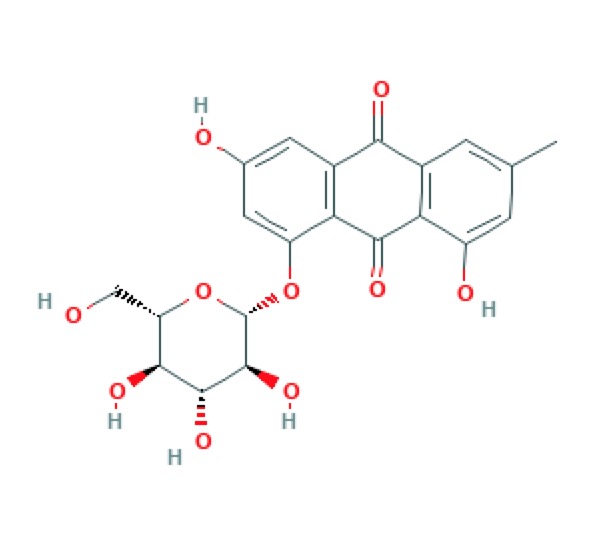 | 44.81 | 0.8 | *Rhei Radix et Rhizoma* |
| MOL002297 | Daucosterol_qt | 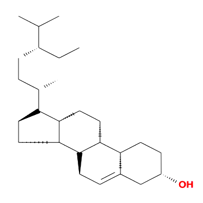 | 35.89 | 0.7 | *Rhei Radix et Rhizoma* |
| MOL000471 | aloe-emodin | 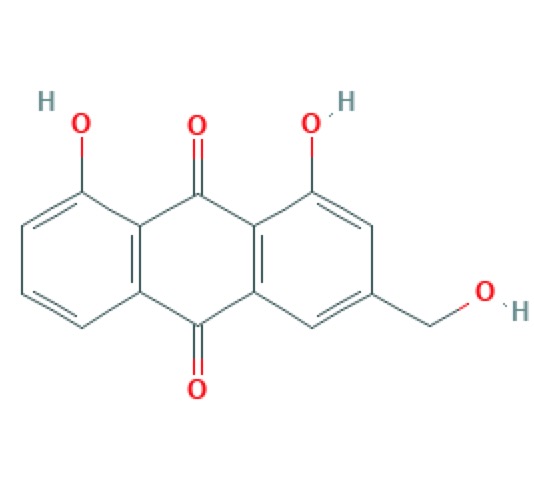 | 83.38 | 0.24 | *Rhei Radix et Rhizoma* |
| MOL000096 | (-)-catechin | 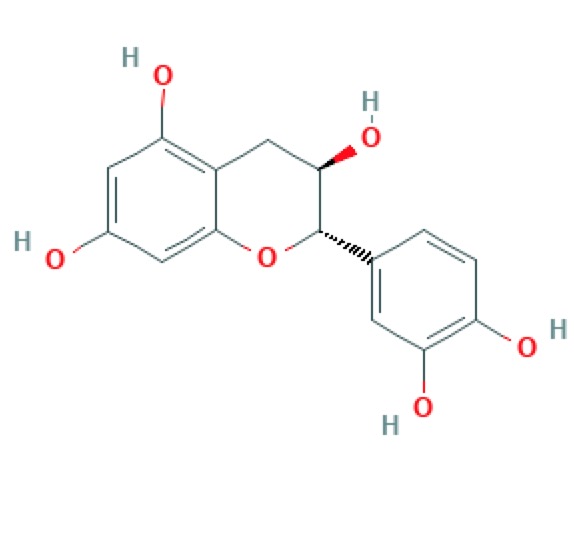 | 49.68 | 0.24 | *Rhei Radix et Rhizoma* |
| MOL001406 | crocetin | 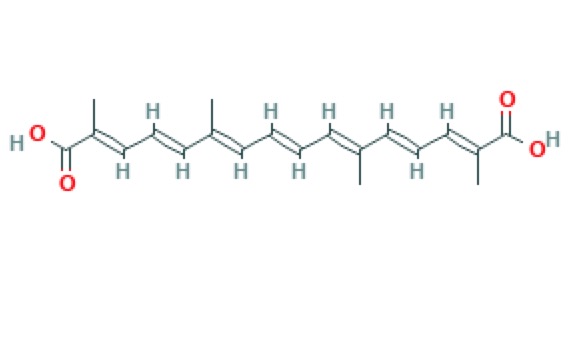 | 35.3 | 0.26 | *Gardeniae Fructus* |
| MOL001941 | Ammidin | 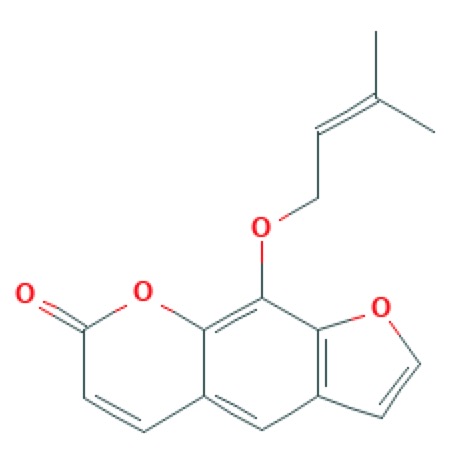 | 34.55 | 0.22 | *Gardeniae Fructus* |
| MOL004561 | Sudan III | 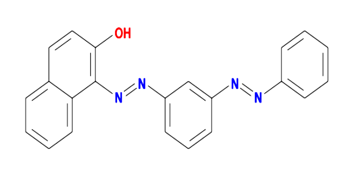 | 84.07 | 0.59 | *Gardeniae Fructus* |
| MOL000098 | quercetin | 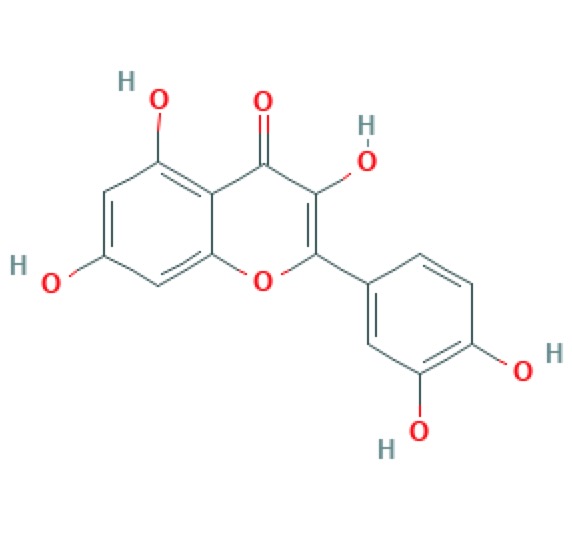 | 46.43 | 0.28 | *Gardeniae Fructus*  *Artemisiae Scopariae Herba* |
| MOL000422 | kaempferol | 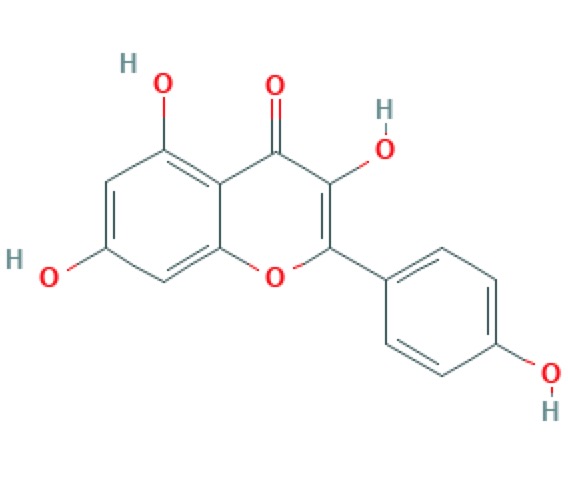 | 41.88 | 0.24 | *Gardeniae Fructus* |
| MOL000449 | Stigmasterol | 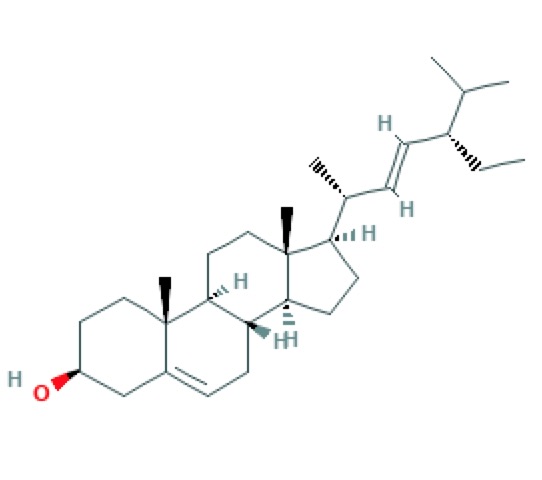 | 43.83 | 0.76 | *Gardeniae Fructus* |
| MOL001494 | Mandenol | 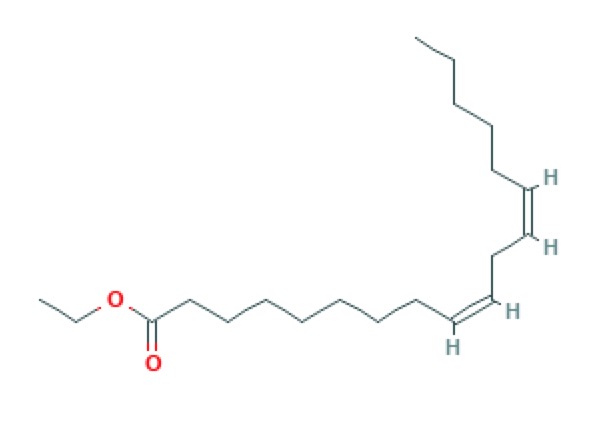 | 42 | 0.19 | *Gardeniae Fructus* |
| MOL001942 | isoimperatorin | 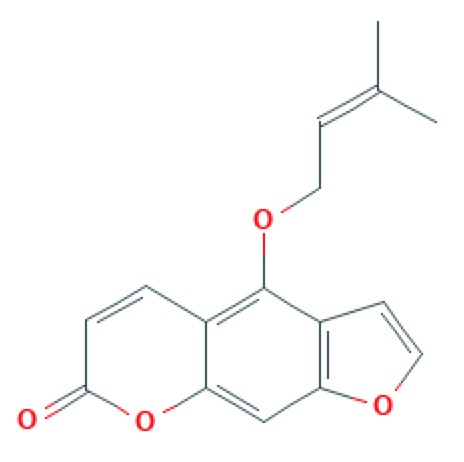 | 45.46 | 0.23 | *Gardeniae Fructus* |
| MOL002883 | Ethyl oleate (NF) | 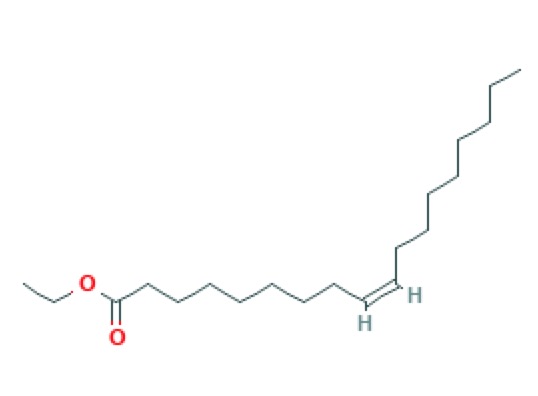 | 32.4 | 0.19 | *Gardeniae Fructus* |
| MOL003095 | 5-hydroxy-7-methoxy-2-(3,4,5-trimethoxyphenyl) chromone | 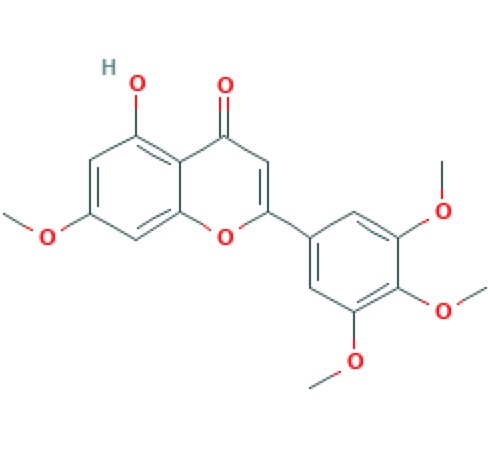 | 51.96 | 0.41 | *Gardeniae Fructus* |
| MOL007245 | 3-Methylkempferol | 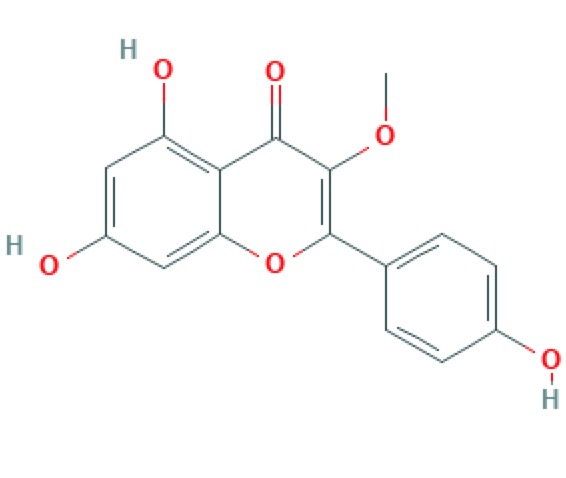 | 60.16 | 0.26 | *Gardeniae Fructus* |
| MOL000354 | isorhamnetin | 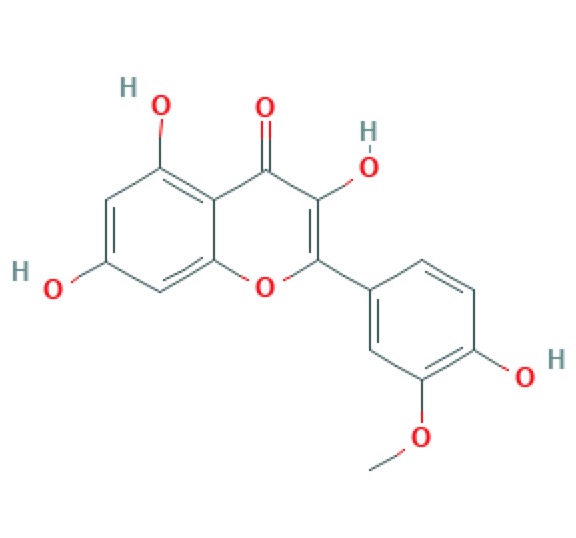 | 49.6 | 0.31 | *Artemisiae Scopariae Herba* |
| MOL000358 | beta-sitosterol | 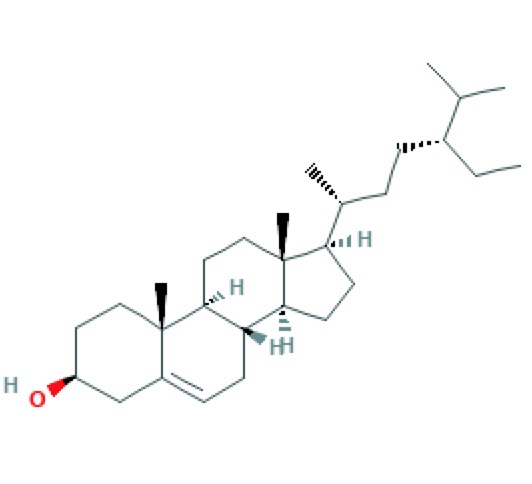 | 36.91 | 0.75 | *Artemisiae Scopariae Herba*  *Gardeniae Fructus*  *Rhei Radix et Rhizoma* |
| MOL004609 | Areapillin | 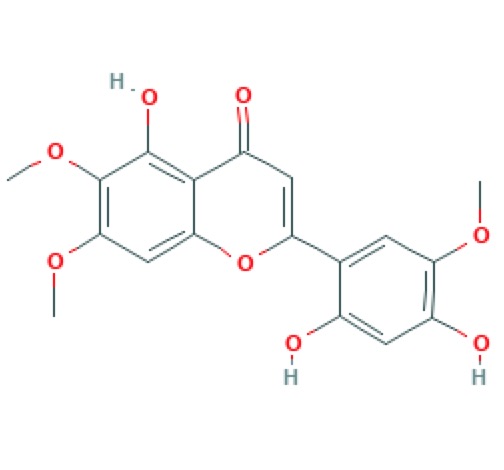 | 48.96 | 0.41 | *Artemisiae Scopariae Herba* |
| MOL005573 | Genkwanin | 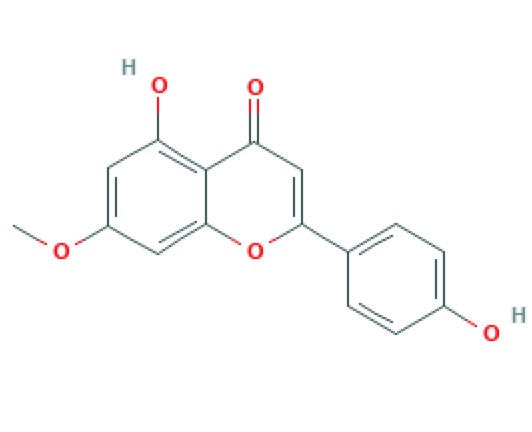 | 37.13 | 0.24 | *Artemisiae Scopariae Herba* |
| MOL007274 | Skrofulein | 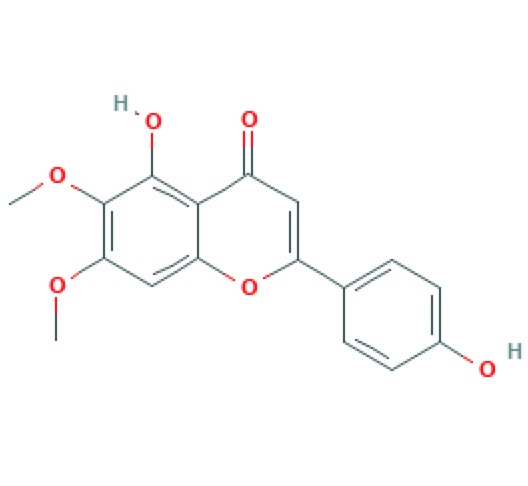 | 30.35 | 0.3 | *Artemisiae Scopariae Herba* |
| MOL008039 | Isoarcapillin | 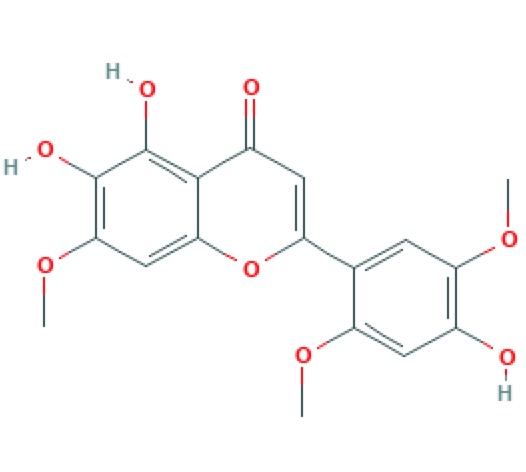 | 57.4 | 0.41 | *Artemisiae Scopariae Herba* |
| MOL008040 | Eupalitin | 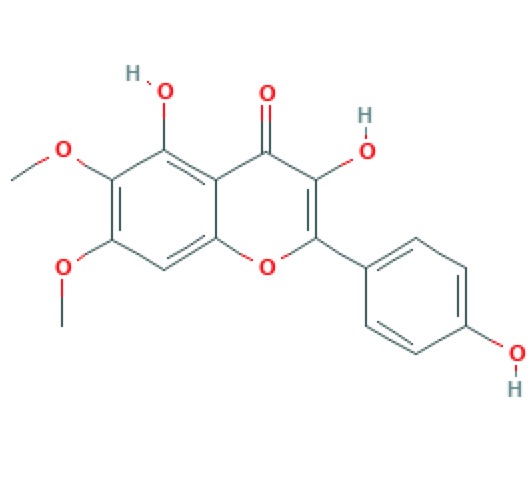 | 46.11 | 0.33 | *Artemisiae Scopariae Herba* |
| MOL008041 | Eupatolitin | 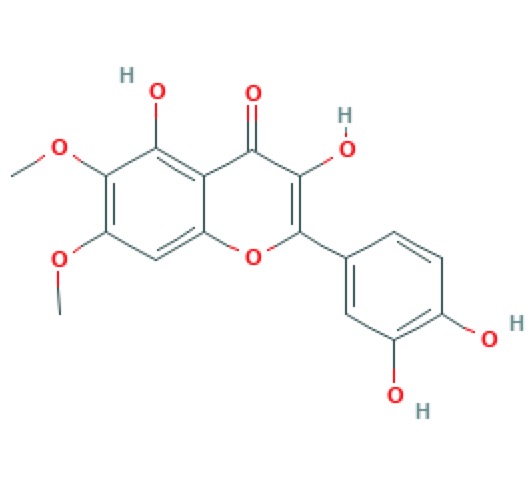 | 42.55 | 0.37 | *Artemisiae Scopariae Herba* |
| MOL008043 | capillarisin | 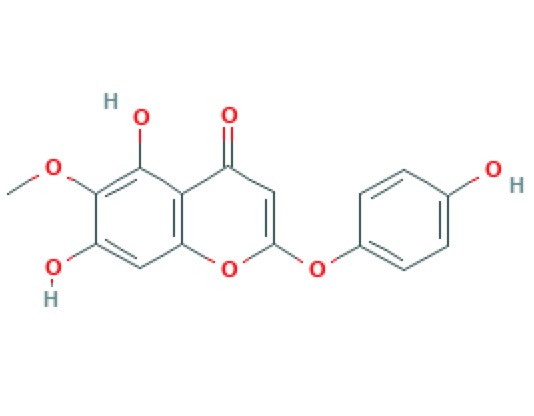 | 57.56 | 0.31 | *Artemisiae Scopariae Herba* |
| MOL008045 | 4'-Methylcapillarisin | 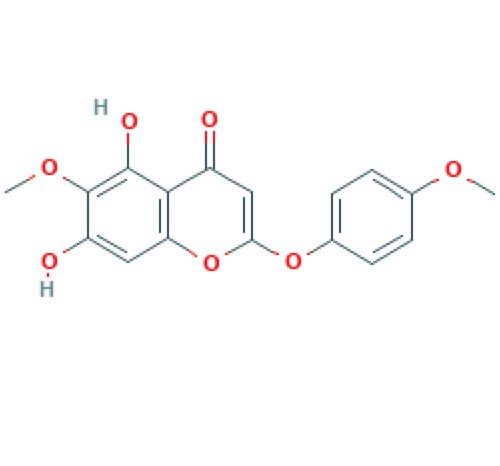 | 72.18 | 0.35 | *Artemisiae Scopariae Herba* |
| MOL008046 | Demethoxycapillarisin | 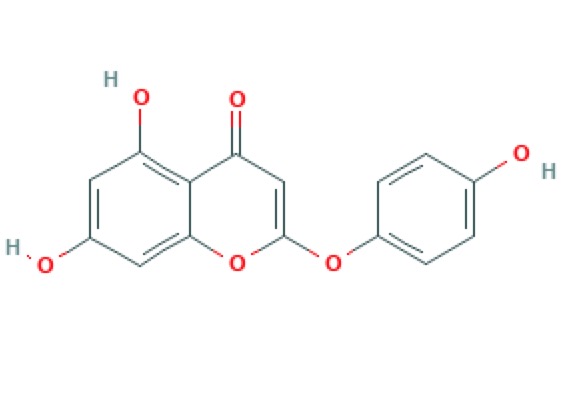 | 52.33 | 0.25 | *Artemisiae Scopariae Herba* |
| MOL008047 | Artepillin A | 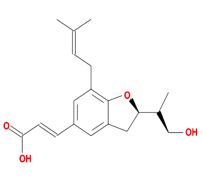 | 68.32 | 0.24 | *Artemisiae Scopariae Herba* |

Table S2: List of 39 proteins related to hepatitis C

| Gene | Protein | Source |
| --- | --- | --- |
| FGFR2 | Fibroblast growth factor receptor 2 | TTD |
| MPL | Thrombopoietin receptor | TTD |
| DGAT1 | Diacylglycerol O-acyltransferase 1 | TTD |
| IFNAR2 | Interferon alpha/beta receptor 2 | TTD |
| IFNA2 | Interferon alpha-2 | TTD |
| TLR7 | Toll-like receptor 7 | TTD |
| IFNA8 | Interferon alpha-8 | TTD |
| MMP2 | 72 kDa type IV collagenase | TTD |
| MMP1 | Interstitial collagenase | TTD |
| SSTR4 | Somatostatin receptor type 4 | TTD |
| TLR9 | Toll-like receptor 9 | TTD |
| IFNAR1 | Interferon alpha/beta receptor 1 | TTD |
| IFNW1 | Interferon omega-1 | TTD |
| IFNA5 | Interferon alpha-5 | TTD |
| MMP9 | Matrix metalloproteinase-9 | TTD |
| MMP13 | Collagenase 3 | TTD |
| IFNL3 | Interferon lambda-3 | pharmGKB |
| IFNL4 | Interferon lambda-4 | pharmGKB |
| HLA-B | HLA class I histocompatibility antigen | pharmGKB |
| CYP24A1 | 1,25-dihydroxyvitamin D (3) 24-hydroxylase | pharmGKB |
| BCL2 | Apoptosis regulator Bcl-2 | pharmGKB |
| SLCA28A2 |  | pharmGKB |
| HLA-C | HLA class I histocompatibility antigen | pharmGKB |
| OASL | 2'-5'-oligoadenylate synthase-like protein | pharmGKB |
| ITPA | Inosine triphosphate pyrophosphatase, ITPase | pharmGKB |
| VDR | Vitamin D3 receptor | pharmGKB |
| SLC29A1 | Equilibrative nucleoside transporter 1 | pharmGKB |
| IDO1 | Indoleamine 2,3-dioxygenase 1 | pharmGKB |
| IL18 | Interleukin-18 | pharmGKB |
| LDLR | Low-density lipoprotein receptor | pharmGKB |
| EGFR | Epidermal growth factor receptor | pharmGKB |
| CYP27B1 | 25-hydroxyvitamin D-1 alpha hydroxylase | pharmGKB |
| CYP2R1 | Vitamin D 25-hydroxylase | pharmGKB |
| CXCL10 | C-X-C motif chemokine 10 | pharmGKB |
| SCARB1 | Scavenger receptor class B member 1 | pharmGKB |
| FTO | Alpha-ketoglutarate-dependent dioxygenase FTO | pharmGKB |
| IL6 | Interleukin-6 | pharmGKB |
| SLC6A4 | Sodium-dependent serotonin transporter | pharmGKB |
| MICB | MHC class I polypeptide-related sequence B | pharmGKB |

Table S3**:** Module 1 GO enrichment entry

| Category | Term | Name | Count | FDR |
| --- | --- | --- | --- | --- |
| MF | GO:0004197 | cysteine-type endopeptidase activity | 8 | 0.000000000 |
| MF | GO:0097153 | cysteine-type endopeptidase activity involved in apoptotic process | 6 | 0.000000001 |
| MF | GO:0005123 | death receptor binding | 5 | 0.000001675 |
| MF | GO:0097200 | cysteine-type endopeptidase activity involved in execution phase of apoptosis | 3 | 0.003302038 |
| MF | GO:0002020 | protease binding | 5 | 0.003551856 |
| MF | GO:0035877 | death effector domain binding | 3 | 0.006599804 |
| MF | GO:0042802 | identical protein binding | 8 | 0.006994808 |
| MF | GO:0005515 | protein binding | 19 | 0.007971296 |
| CC | GO:0031265 | CD95 death-inducing signaling complex | 5 | 0.000000010 |
| CC | GO:0031264 | death-inducing signaling complex | 5 | 0.000000023 |
| CC | GO:0005829 | cytosol | 16 | 0.000003492 |
| CC | GO:0097342 | ripoptosome | 4 | 0.000027444 |
| BP | GO:0006915 | apoptotic process | 17 | 0.000000000 |
| BP | GO:0006919 | activation of cysteine-type endopeptidase activity involved in apoptotic process | 11 | 0.000000000 |
| BP | GO:0042981 | regulation of apoptotic process | 13 | 0.000000000 |
| BP | GO:1902041 | regulation of extrinsic apoptotic signaling pathway via death domain receptors | 7 | 0.000000000 |
| BP | GO:1902042 | negative regulation of extrinsic apoptotic signaling pathway via death domain receptors | 7 | 0.000000001 |
| BP | GO:0097192 | extrinsic apoptotic signaling pathway in absence of ligand | 7 | 0.000000001 |
| BP | GO:0097296 | activation of cysteine-type endopeptidase activity involved in apoptotic signaling pathway | 6 | 0.000000001 |
| BP | GO:0008625 | extrinsic apoptotic signaling pathway via death domain receptors | 7 | 0.000000002 |
| BP | GO:0043065 | positive regulation of apoptotic process | 9 | 0.000000470 |
| BP | GO:0097194 | execution phase of apoptosis | 5 | 0.000002212 |
| BP | GO:0071260 | cellular response to mechanical stimulus | 6 | 0.000012746 |
| BP | GO:0097190 | apoptotic signaling pathway | 6 | 0.000012746 |
| BP | GO:0008635 | activation of cysteine-type endopeptidase activity involved in apoptotic process by cytochrome c | 4 | 0.000114719 |
| BP | GO:0043525 | positive regulation of neuron apoptotic process | 5 | 0.000147303 |
| BP | GO:0043123 | positive regulation of I-kappaB kinase/NF-kappaB signaling | 6 | 0.000781936 |
| BP | GO:0032496 | response to lipopolysaccharide | 6 | 0.000856876 |
| BP | GO:0006921 | cellular component disassembly involved in execution phase of apoptosis | 4 | 0.001801825 |
| BP | GO:0036462 | TRAIL-activated apoptotic signaling pathway | 3 | 0.004313920 |
| BP | GO:0090200 | positive regulation of release of cytochrome c from mitochondria | 4 | 0.004417349 |
| BP | GO:1900740 | positive regulation of protein insertion into mitochondrial membrane involved in apoptotic signaling pathway | 4 | 0.005467131 |
| BP | GO:0046677 | response to antibiotic | 4 | 0.006670071 |
| BP | GO:0051402 | neuron apoptotic process | 4 | 0.007332139 |
| BP | GO:2001244 | positive regulation of intrinsic apoptotic signaling pathway | 4 | 0.007332139 |

Table S4**:** Module 2 GO enrichment entry

| Category | Term | Name | Count | FDR |
| --- | --- | --- | --- | --- |
| MF | GO:0016303 | 1-phosphatidylinositol-3-kinase activity | 5 | 0.000051900 |
| MF | GO:0046934 | phosphatidylinositol-4,5-bisphosphate 3-kinase activity | 5 | 0.000232000 |
| MF | GO:0046875 | ephrin receptor binding | 4 | 0.001549772 |
| MF | GO:0005088 | Ras guanyl-nucleotide exchange factor activity | 5 | 0.002798906 |
| CC | GO:0035032 | phosphatidylinositol 3-kinase complex, class III | 3 | 0.006644506 |
| BP | GO:0007173 | epidermal growth factor receptor signaling pathway | 8 | 0.000000000 |
| BP | GO:0038128 | ERBB2 signaling pathway | 7 | 0.000000001 |
| BP | GO:0048015 | phosphatidylinositol-mediated signaling | 8 | 0.000000003 |
| BP | GO:0046854 | phosphatidylinositol phosphorylation | 6 | 0.000019700 |
| BP | GO:0036092 | phosphatidylinositol-3-phosphate biosynthetic process | 5 | 0.000119000 |
| BP | GO:0038095 | Fc-epsilon receptor signaling pathway | 6 | 0.000485000 |
| BP | GO:0008284 | positive regulation of cell proliferation | 7 | 0.002487663 |
| BP | GO:0000165 | MAPK cascade | 6 | 0.003269796 |
| BP | GO:0050900 | leukocyte migration | 5 | 0.004730441 |
| BP | GO:0042059 | negative regulation of epidermal growth factor receptor signaling pathway | 4 | 0.005623498 |
| BP | GO:0043547 | positive regulation of GTPase activity | 7 | 0.007586245 |
| BP | GO:0045740 | positive regulation of DNA replication | 4 | 0.009012506 |

Table S5**:** Module 1 KEGG enrichment entry

| Category | Term | Name | Count | FDR |
| --- | --- | --- | --- | --- |
| KEGG | hsa04210 | Apoptosis | 16 | 0.000000000 |
| KEGG | hsa04668 | TNF signaling pathway | 7 | 0.000088800 |
| KEGG | hsa04115 | p53 signaling pathway | 6 | 0.000310000 |
| KEGG | hsa05161 | Hepatitis B | 7 | 0.000544000 |
| KEGG | hsa05200 | Pathways in cancer | 9 | 0.000928000 |
| KEGG | hsa05010 | Alzheimer's disease | 7 | 0.001297441 |
| KEGG | hsa05014 | Amyotrophic lateral sclerosis (ALS) | 5 | 0.004310509 |
| KEGG | hsa04650 | Natural killer cell mediated cytotoxicity | 6 | 0.006173388 |

Table S6**:** Module 2 KEGG enrichment entry

| Category | Term | Name | Count | FDR |
| --- | --- | --- | --- | --- |
| KEGG | hsa04012 | ErbB signaling pathway | 9 | 0.000000003 |
| KEGG | hsa05214 | Glioma | 8 | 0.000000033 |
| KEGG | hsa05220 | Chronic myeloid leukemia | 8 | 0.000000069 |
| KEGG | hsa05213 | Endometrial cancer | 7 | 0.000000742 |
| KEGG | hsa05223 | Non-small cell lung cancer | 7 | 0.000001180 |
| KEGG | hsa04068 | FoxO signaling pathway | 8 | 0.000005790 |
| KEGG | hsa04014 | Ras signaling pathway | 9 | 0.000007130 |
| KEGG | hsa05215 | Prostate cancer | 7 | 0.000019000 |
| KEGG | hsa05200 | Pathways in cancer | 10 | 0.000024600 |
| KEGG | hsa04915 | Estrogen signaling pathway | 7 | 0.000038700 |
| KEGG | hsa05231 | Choline metabolism in cancer | 7 | 0.000043700 |
| KEGG | hsa04510 | Focal adhesion | 8 | 0.000115000 |
| KEGG | hsa04722 | Neurotrophin signaling pathway | 7 | 0.000123000 |
| KEGG | hsa04151 | PI3K-Akt signaling pathway | 9 | 0.000197000 |
| KEGG | hsa05160 | Hepatitis C | 7 | 0.000228000 |
| KEGG | hsa04664 | Fc epsilon RI signaling pathway | 6 | 0.000255000 |
| KEGG | hsa04910 | Insulin signaling pathway | 7 | 0.000284000 |
| KEGG | hsa04917 | Prolactin signaling pathway | 6 | 0.000317000 |
| KEGG | hsa04066 | HIF-1 signaling pathway | 6 | 0.001443295 |
| KEGG | hsa05205 | Proteoglycans in cancer | 7 | 0.002537832 |
| KEGG | hsa04650 | Natural killer cell mediated cytotoxicity | 6 | 0.004741171 |
| KEGG | hsa05221 | Acute myeloid leukemia | 5 | 0.005664779 |
| KEGG | hsa05230 | Central carbon metabolism in cancer | 5 | 0.009699068 |

Table S7: List of GO enrichment results to the YCHD potential target associated with hepatitis C.

| Category | Term | Name | Count | FDR |
| --- | --- | --- | --- | --- |
| BP | GO:0043066 | negative regulation of apoptotic process | 7 | 0.000226556 |
| BP | GO:0008284 | positive regulation of cell proliferation | 6 | 0.009313397 |
| BP | GO:0051384 | response to glucocorticoid | 4 | 0.012835701 |
| BP | GO:0042060 | wound healing | 4 | 0.024012782 |

Table S8: List of pathway enrichment results to the YCHD potential target associated with hepatitis C.

| Term | Name | Count | FDR |
| --- | --- | --- | --- |
| hsa05200 | Pathways in cancer | 10 | 0.000000339 |
| hsa04151 | PI3K-Akt signaling pathway | 7 | 0.006545183 |
| hsa04068 | FoxO signaling pathway | 6 | 0.001260564 |
| hsa05152 | Tuberculosis | 6 | 0.005001832 |
| hsa05161 | Hepatitis B | 6 | 0.001865811 |
| hsa04210 | Apoptosis | 5 | 0.002168446 |
| hsa05212 | Pancreatic cancer | 5 | 0.002625315 |
| hsa05215 | Prostate cancer | 5 | 0.008873028 |
| hsa04066 | HIF-1 signaling pathway | 5 | 0.012557352 |
| hsa04668 | TNF signaling pathway | 5 | 0.019331494 |
| hsa05145 | Toxoplasmosis | 5 | 0.021572678 |
| hsa05219 | Bladder cancer | 4 | 0.036118154 |
